# The Role of Micro RNA and Long-Non-Coding RNA in Osteoporosis

**DOI:** 10.3390/ijms21144886

**Published:** 2020-07-10

**Authors:** Nai-Yu Ko, Li-Ru Chen, Kuo-Hu Chen

**Affiliations:** 1 Department of Physical Medicine and Rehabilitation, Mackay Memorial Hospital, Taipei 104, Taiwan; Naiyuko@gmail.com (N.-Y.K.); gracealex168@gmail.com (L.-R.C.); 2 Department of Mechanical Engineering, National Chiao-Tung University, Hsinchu 300, Taiwan; 3 Department of Obstetrics and Gynecology, Taipei Tzu-Chi Hospital, The Buddhist Tzu-Chi Medical Foundation, Taipei 231, Taiwan; 4 School of Medicine, Tzu-Chi University, Hualien 970, Taiwan

**Keywords:** osteoporosis, micro RNA, miRNA, long-non-coding RNA, lncRNA

## Abstract

Osteoporosis is a major concern worldwide and can be attributed to an imbalance between osteoblastic bone formation and osteoclastic bone resorption due to the natural aging process. Heritable factors account for 60–80% of optimal bone mineralization; however, the finer details of pathogenesis remain to be elucidated. Micro RNA (miRNA) and long-non-coding RNA (lncRNA) are two targets that have recently come into the spotlight due to their ability to control gene expression at the post-transcriptional level and provide epigenetic modification. miRNAs are a class of non-coding RNAs that are approximately 18–25 nucleotides long. It is thought that up to 60% of human protein-coding genes may be regulated by miRNAs. They have been found to regulate gene expression that controls osteoblast-dependent bone formation and osteoclast-related bone remodeling. lncRNAs are highly structured RNA transcripts longer than 200 nucleotides that do not translate into proteins. They have very complex secondary and tertiary structures and the same degradation processes as messenger RNAs. The fact that they have a rapid turnover is due to their sponge function in binding the miRNAs that lead to a degradation of the lncRNA itself. They can act as signaling, decoy, and framework molecules, or as primers. Current evidence suggests that lncRNAs can act as chromatin and transcriptional as well as post-transcriptional regulators. With regards to osteoporosis, lncRNA is thought to be involved in the proliferation, apoptosis, and inflammatory response of the bone. This review, which is based on a systematic appraisal of the current literature, provides current molecular and genetic opinions on the roles of miRNAs and lncRNAs in osteoporosis. Further research into the epigenetic modification and the regulatory roles of these molecules will bring us closer to potential disease-modifying treatment for osteoporosis. However, more issues regarding the detailed actions of miRNAs and lncRNAs in osteoporosis remain unknown and controversial and warrant future investigation.

## 1. Overview

Osteoporosis is increasing in prevalence around the world due to the aging population. Although osteoporosis can be attributed to an imbalance between osteoblastic bone formation and osteoclastic bone resorption [1] as part of the natural aging process, the finer details of pathogenesis remain to be elucidated. Current treatment methods, including bisphosphonates, hormone replacement therapy, and immunotherapy, all carry the risk of side effects related to their mechanisms of action. Therefore, novel therapies are being developed. Further understanding of the underlying pathophysiology is paramount to developing these new therapies.

Micro RNA (miRNA) and long-non-coding RNA (lncRNA) are two such targets that have recently come into the spotlight due to their ability to control gene expression at the post-transcriptional level, providing epigenetic modification [2,3]. miRNAs are a class of non-coding RNAs that are approximately 18–25 nucleotides long [4]. It is thought that up to 60% of human protein-coding genes may be regulated by miRNAs [2,5]. They bind to the 3-untranslated regions (3-UTR) of target genes, leading to messenger RNA (mRNA) degradation and transcription inhibition [2,6]. The process of miRNA regulation is complex, as each miRNA binds to a number of targets, and several miRNAs target the same mRNA [2]. They have been found to regulate most biological processes, including cell development, differentiation, proliferation, metabolism, and cell cycle regulation [2]. They have also been found to regulate gene expression that controls osteoblast-dependent bone formation and osteoclast-related bone remodeling [3,7].

lncRNAs are highly structured RNA transcripts longer than 200 nucleotides that do not translate into proteins [8]. In fact, lncRNAs have very complex secondary and tertiary structures and the same degradation processes as mRNAs. The fact that they have a rapid turnover is due to their sponge function in binding the miRNAs that lead to a degradation of the lncRNA itself [6,9]. They can act as signaling, decoy, and framework molecules, or as primers [9]. Current evidence suggests that lncRNAs can act as chromatin and transcriptional as well as post-transcriptional regulators [8]. With regards to osteoporosis, lncRNA is thought to be involved in the proliferation, apoptosis, and inflammatory response of the bone [10].

The interaction between miRNAs and lncRNAs is also of current interest. Studies have attempted to link lncRNAs, miRNAs, and mRNAs together in a complex network, such as Hao et al.’s systematic analysis using the mandibles from ovariectomized mice [11]. Fei and colleagues performed a small study in five Chinese women to identify the key lncRNAs in postmenopausal osteoporosis (PMOP) through RNA sequencing [12]. After identifying various differentially expressed mRNAs (DEmRNAs) and differentially expressed lncRNAs (DElncRNAs), they constructed a DElncRNA-DEmRNA co-expression network [12]. In a larger study, Zhou et al. identified lncRNAs in 73 Caucasian women with PMOP and established an mRNA/lncRNA co-expression network [13]. The shared goal of the above studies was to provide a foundation for future investigations of lncRNAs in PMOP and help to develop biomarkers and drugs. In this review, we aim to summarize the current evidence on the actions of various miRNA and lncRNA. IsomiRNAs are grouped together. 

## 2. The Role of Micro RNA (miRNA) and Long-Non-Coding RNA (lncRNA) in Osteoporosis

### 2.1. Micro RNA (miRNA)

#### 2.1.1. miR-9-5p

miR-9-5p inhibits osteogenesis and promotes adipogenesis via directly binding to Wnt3a [14]. It also promotes osteoclastogenesis [15]. In a study of 30 osteoporosis patients, miR-9-5p was found to be more highly expressed in the serum of osteoporosis patients compared to healthy controls [14].

#### 2.1.2. miR-21

miR-21 has been found to promote osteoclastogenesis. Through a positive feedback loop that involves programmed cell death, miR-21 is upregulated by osteoclastogenesis factor c-Fos and then promotes RANKL (receptor activator of nuclear factor-κB ligand)-induced osteoclastogenesis [1]. Jiang and colleagues found that miR-21 targeted SMAD7 and inhibited osteogenesis [16]. A review by Cheng et al. has concluded that studies consistently showed a positive role of miR-21 in osteoclastogenesis [17]. miR-21 was found to be increased in the serum and bone tissue of osteoporotic patients [18]. However, miR-21-5p expression has been found to be significantly lower among osteoporotic/osteopenic women with vertebral fractures [19]. The action of miR-21 is doubtful as it appears to be both up- and downregulated in postmenopausal osteoporosis trials [18,19,20,21,22]. In some in vitro studies, it is reported that it promotes osteogenesis, while in others, it inhibits osteoclastogenesis [23,24]. Therefore, further studies are needed to determine whether it can be used as a clinical biomarker for osteoporosis.

#### 2.1.3. miR-29

Decreased miR-29 is a potential marker of osteoporosis. Lower serum miR-29 levels were associated with vertebral fractures in postmenopausal women [25]. In osteoblast-specific miR-29a transgenic mice, overexpression of miR-29a increased bone formation and decreased osteoclastic resorption by inhibiting RANKL and CXCL12 (C-X-C Motif Chemokine Ligand 12) expression in osteoblasts [26]. miR-29 is described to act in favoring of osteogenesis and limiting osteoclastogenesis [27,28,29], and it is downregulated in patients with osteoporosis [26,30]. miR-29b-3p, on the other hand, is known to downregulate multiple genes involved in osteoblast formation and is induced in osteoclast formation [31]. Other studies suggest miR-29 has a role in osteoclast differentiation, but reports on its mechanism of action are conflicting [1].

#### 2.1.4. miR-30b-5p

In osteoporotic women, serum levels of miR-30b-5p were low. In vitro studies of osteogenesis have validated that miR-30b-5p targets RUNX2 (runt-related transcription factor 2) and decreases during the late stages of osteoblast differentiation [31].

#### 2.1.5. miR-31

miR-31-3p has been found to inhibit osteoclastic bone resorption through the repression of osteoclast formation [1]. However, other studies have found that miR-31a-5p expression in BMSCs (bone mesenchymal stem cells) increases with age, and increases osteoclastogenesis, thereby contributing to age-related bone loss [5]. miR-31-5p was found to be downregulated in patients with WNT1 osteoporosis, a primary osteoporosis due to heterozygous p.C218G WNT1 mutation [32]. miR-31-5p is known to inhibit WNT signaling, leading to low bone formation and, therefore, the increased risk of bone fractures [32].

#### 2.1.6. miR-100

Levels of miR-100 are increased in the serum and bone tissue of osteoporotic patients [18]. Cheng et al.’s systematic review found that miR-100 inhibited bone formation [17]. Osteoporotic patients had upregulation of miR-100-5p in both osteoblasts and osteoclasts, so it was speculated that an overall increase in expression led to bone loss. miR-100 expression was decreased during osteoblastic differentiation in vitro, and overexpression of miR-100 in MSC inhibited osteogenic differentiation. The role of miR-100 in osteoclastogenesis has not yet been established [17].

#### 2.1.7. miR-103-3p

The levels of serum miR-103-3p are lower in osteoporotic women when compared with healthy controls [31]. In vitro studies of osteogenesis have suggested that miR-103-3p inhibits osteoblast differentiation and proliferation [31].

#### 2.1.8. miR-122-5p

An analysis of 139 serum samples using RT-qPCR (real-time quantitative polymerase chain reaction) showed that lower levels of miR122-5p were found in patients with lower BMD (bone mineral density), and, therefore, may be associated with the development of osteoporosis [33]. This makes it a likely diagnostic biomarker for osteoporosis [33].

#### 2.1.9. miR-124

miR-124 could potentially alleviate the progression of osteoporosis. Studies have shown miR-124 to suppress differentiation and migration of osteoclast precursors, thereby inhibiting osteoclast formation [1]. However, it also inhibits osteogenesis [34]. Similarly, serum miR-124-3p was found to be significantly upregulated in postmenopausal women with low BMD [19]. The authors of this study suggested that a possible explanation would be a compensatory mechanism of bone tissue in response to menopause-induced bone destruction [19].

#### 2.1.10. miR-133 Family

miR-133 increases osteoclastogenesis due to mRNA targeting of the proteins that inhibit osteoclastogenesis [35]. miR-133a is upregulated in osteoporosis. It targets the RUNX2 gene 3′-UTR when overexpressed in an osteoblast cell line, and suppresses alkaline phosphatase (ALP) (a marker of osteoblast formation) production, and, therefore, osteoblast differentiation [1]. Using bioinformatics analysis, three osteoclast-related potential target genes have been identified for miR-133a (CXCL11, CXCR3, and SLC39A1), but the sample size was limited [31]. A new study led by Kocijan found miRNA-133 to be downregulated in the serum of animals receiving zoledronic acid, which suggests a positive effect of bisphosphonates on RUNX2 and thus, bone formation [36]. Cheng et al. also summarized that miR-133a promoted bone resorption and could potentially inhibit bone formation [17]

#### 2.1.11. miR-135a-5p

miR-135a-5p levels were elevated in bone tissue of postmenopausal women with osteoporosis compared with postmenopausal women without osteoporosis [37]. miR-135a-5p was found to be potentially downregulated during osteogenic differentiation [37]. The study also suggested that miR-135a-5p inhibited osteogenic differentiation by targeting RUNX2 directly [37].

#### 2.1.12. miR-146a

Another potential target for treating osteoporosis is miR-146a, found in bone tissue, which can be induced by TNF-a/RANKL treatment, and has been found to inhibit osteoclastogenesis in mouse models [1].

#### 2.1.13. miR-148a

miR-148a overexpression induces osteoclast formation [1]. In an ovariectomized rat model, overexpression of microRNA-148a in the serum was associated with apoptosis and inhibition of cell growth [38]. It significantly reduced the expression of estrogen receptor alpha (ERα), phosphoinositide-3-kinase regulatory subunit 1 (PI3K), and phosphorylated-protein kinase B (AKT) in osteoblasts in vitro [38]. One can infer that suppressing miR-148a could potentially help treat osteoporosis.

#### 2.1.14. miR-155

miR-155 plays an important role in bone destruction, as demonstrated by its involvement in rheumatoid arthritis [1]. It is now known that miR-155 regulates osteoclastogenesis through several essential transcriptional factors, such as inhibiting MITF (microphthalmia-associated transcription factor) [1].

#### 2.1.15. miR-182-5p

In Pan et al.’s animal study, ovariectomized rats treated with alendronate were used to assess the effects of miR-182-5p [39]. They found that in the alendronate treatment group, miR-182-5p was downregulated in the serum, ADCY6 (Adenylate Cyclase 6) was upregulated, and the Rap1/MAPK (mitogen-activated protein kinase) signaling pathway was activated [39]. Then, RT-qPCR and Western blot analysis showed that miR-182-5p inhibited ADCY6 expression and Rap1/MAPK signaling pathway activation, while downregulation of miR-182-5p inhibited cell cycle progression as well as osteoblastic cell apoptosis [39]. Therefore, decreasing miR-182-5p could be a potential goal of osteoporosis treatment.

#### 2.1.16. miR-194-5p

In whole blood lysates of postmenopausal Chinese women, miR-194-5p was found to be upregulated by over five-fold, and could potentially be used to discriminate against osteopenia and osteoporosis [5].

#### 2.1.17. miR-200a-3p

Blood was collected from 30 postmenopausal women, and the miR-200a-3p level was found to be higher in the serum of these patients compared with controls [40]. High levels of miR-200a-3p suppressed osteogenic differentiation of BMSCs [40].

#### 2.1.18. miR-203a

miR-203a is found to be upregulated in the bone tissue from postmenopausal women with a history of low-traumatic fractures and slows osteoblast differentiation [36].

#### 2.1.19. miR-214-5p

An in vitro study showed that miR-214-5p overexpression promoted adipogenic differentiation, thereby promoting osteoporosis progression [41]. miR-214-5p was found to promote adipogenic differentiation of human BMSCs through the regulation of the TGF (transforming growth factor)-β/Smad2/COL4A1 (collagen type IV α1 chain) signaling pathway and would downregulate the expression of ALP, RUNX2, osteocalcin, collagen α-1 (I) chain (COL1A1) mRNA, TGF-β, phosphorylated (p)-Smad2, and COL4A1 protein [41].

#### 2.1.20. miR-221

Bony fragments extracted from 12 women with PMOP fractures undergoing hip replacements showed that miR-221 was underexpressed compared to the 12 healthy controls (osteoarthritis without osteoporosis) [42]. miR-221 was found to inhibit osteogenic inhibition by negatively regulating RUNX2 expression [42].

#### 2.1.21. miR-223

Upregulation of miR-223 is thought to inhibit osteoclast differentiation [1]. miR-223-5p was found to be upregulated in the serum of osteoporotic patients with hip fractures compared to nonosteoporotic women [18]. However, miR-223-5p was not associated with incident osteoporotic fractures in a large cohort of 217 women [43], causing some doubt on its clinical use.

#### 2.1.22. miR-338 Cluster

In Guo et al.’s study, miR-338-3p was found to be decreased during osteoblast differentiation, and that miR-338-3p knockout upregulated RUNX2 at the mRNA level [44]. In a small sample (n = 15) of PMOP patients, significantly increased miR-338 levels in the serum were found [45]. Ovariectomized mice also had increased levels of miR-338, and this was detected earlier than the decrease in bone density measured by micro-CT [45]. An estrogen-dependent Runx2/Sox4 (SRY-Box Transcription Factor 4)/miR-338 positive feedback loop could afford to regulate osteoblast differentiation. The use of a miR-388 inhibitor in the mice significantly prevented osteoporosis after an ovariectomy [45].

#### 2.1.23. miR-365

In glucocorticoid-induced osteoporosis (GIOP) mice, levels of miR-365 were found to be suppressed in bone tissue [46]. MMP-9 (matrix metalloproteinase-9) is produced by osteoclasts and assists in the degradation of the extracellular matrix [46]. Bioinformatics analysis suggests that activation of miR-365 suppresses MMP-9 [46]. Therefore, activation of miR-365 could be a novel target of osteoporosis treatment.

#### 2.1.24. miR-410

In 26 postmenopausal women with osteoporosis, and also in ovariectomized mice, there is elevated miR-410 and reduced BMP-2 (bone morphogenetic protein 2) in serum samples [47]. Bioinformatics analysis has shown that miR-410 binds to BMP-2 and regulates its expression. It is known that BMP-2 can control miRNA expression, including the switch between bone and muscle differentiation [3]. However, this study had a small sample size with a lack of genetic diversity, and further studies are needed to help elucidate the role of miR-410 in osteoporosis.

#### 2.1.25. miR-422a

miR-422a is significantly upregulated in the circulating monocytes of postmenopausal women with low BMD in a study by Cao et al. [48]. Using bioinformatics analysis, five potential osteoclast-related target genes were identified for miR-422a (CBL, CD226, IGF1, PAG1, TOB2), but the sample size was limited [31]. miR-422a may stimulate osteoclastogenesis, but further research is required to elucidate its mechanism of action [48].

#### 2.1.26. miR-449b-5p

In vivo findings suggest that miR-449 overexpression could inhibit the osteogenic differentiation of BMSCs by binding directly to the 3-UTR terminus of SATB2 (Special AT-rich sequence-binding protein 2) and suppressing SATB2 [49]. Human studies are required to determine the serum levels of miR-449b-5p in PMOP.

#### 2.1.27. miR-503

miR-503 expression was found to be markedly reduced in PMOP [1]. miR-503 overexpression in human peripheral blood monocytes led to inhibition of RANKL-induced osteoclast differentiation [1].

#### 2.1.28. miR-543

In ovariectomized rats, overexpression of miRNA-543 was found to significantly suppress cell growth and promote apoptosis in osteoblasts [50]. In addition, miRNA-543 upregulation inhibited YAF-2 (YY1-associated factor 2) expression in osteoblasts [50]. The effects of miRNA-543 were further enhanced by YAF-2 knockdown [50]. These findings suggest that inhibiting miRNA-543 could potentially protect osteoblasts against ovariectomy-induced osteoporosis through the AKT/p38 MAPK signaling pathway and targeting YAF2 [50]. This could provide a potential therapeutic target for PMOP.

#### 2.1.29. miR-579-3p

Micro RNA-579-3P expression in the serum of osteoporotic patients was significantly higher than those of normal controls, and this inhibited osteogenic differentiation of human BMSCs [51].

#### 2.1.30. miR-874

In osteoporotic rats, there was inactivation of miR-874 and SUFU (suppressor of fused gene) overexpression in bone tissue. Both upregulation of miR-874 and downregulation of SUFU were found to promote osteoblast proliferation [52]. Human studies are required to establish the role of miR-874 in PMOP.

#### 2.1.31. miR-1297

miR-1297, highly expressed in osteoporosis, has been found to inhibit osteogenic differentiation of human BMSCs [53]. It affects the Wnt signaling pathway; its direct target is WNT5A [53]. An in vitro study has shown that levels of miR-1297 decreased after osteogenic induction [53], providing a novel way to monitor PMOP treatment. 

#### 2.1.32. miR-2861

Decreased miR-2861 may contribute to the pathogenesis of osteoporosis. miR-2861 was identified in primary mouse osteoblasts, and it was found to promote osteoblast differentiation by suppressing histone deacetylase 5 (HDAC5) expression at the post-transcriptional level [54]. HDAC5 is an enhancer of RUNX2 degradation [54]. Overexpression of miR-2861 also enhances BMP2-induced osteoblastogenesis, and inhibition attenuates it [54]. In vivo silencing of miR-2861 in mice was found to reduce RUNX2 protein expression, which led to inhibited bone formation and decreased bone mass [54]. Surprisingly, miR-2861 was found to be conserved in humans, and a homozygous mutation that blocked miR-2861 expression was shown to cause primary osteoporosis in two related adolescents [54]. Therefore, this study shows that miR-2861 plays an important physiological role in osteoblast differentiation. Serum levels of miR-2861 were found to be higher in postmenopausal women with low BMD, which could reflect a compensatory mechanism of the human body towards menopause-induced bone destruction [19].

Table 1 and Table 2 provide a brief overview of the molecules listed.

### 2.2. Long-Non-Coding RNA (lncRNA)

**Figure 1:**
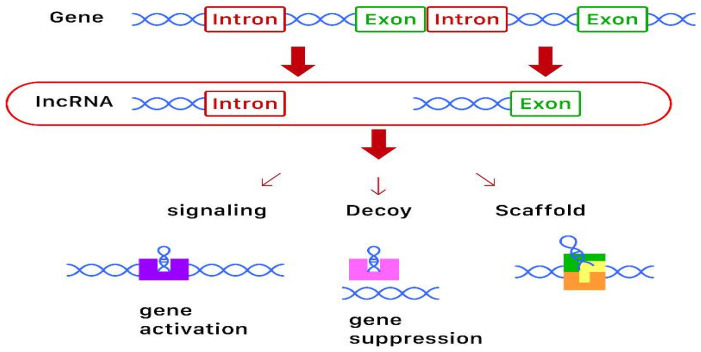
Diagram of the biosynthesis and function of long-non-coding RNAs (lncRNAs). lncRNAs are derived from genes, some containing introns and some containing exons. These lncRNAs then act as signaling (for gene activation), decoy (for gene suppression), or scaffold molecules to exert epigenetic modification.

lncRNAs are highly structured RNA transcripts longer than 200 nucleotides that do not translate into proteins [8]. They have very complex secondary and tertiary structures and the same degradation processes as mRNAs. Although similar to mRNAs, lncRNAs degrade more easily due to their sponge function in binding the miRNAs that lead to a degradation of the lncRNA itself [6,9]. They can act as signaling, decoy, and framework molecules, or as primers. Current evidence suggests that lncRNAs can act as chromatin and transcriptional as well as post-transcriptional regulators. With regards to osteoporosis, lncRNA is thought to be involved in the proliferation, apoptosis, and inflammatory response of the bone. A diagram of the biosynthesis, action, and function of lncRNAs is shown in Figure 1.

#### 2.2.1. lncRNA-ANCR

There is evidence that lncRNA-ANCR (anti-differentiation non-coding RNA), also known as DANCR (differentiation antagonizing non-protein coding RNA), promotes osteoporosis [9]. QRT-PCR detection showed that lncRNA-ANCR was increased in the osteoblast group in the PMOP mice model [55]. When ANCR was silenced through the transfection of postmenopausal mice, their osteoblast cells showed decreased apoptosis and increased proliferation [55]. It is also upregulated in the blood mononuclear cells in postmenopausal women with low BMD and is found to promote IL6 and TNF-α expression [56]. As IL6 and TNF-α are inflammatory markers involved in osteoclastogenesis [56], it can be inferred that DANCR could serve as a potential biomarker for osteoporosis.

#### 2.2.2. lncRNA BMNCR

lncRNA BMNCR (bone marrow associated non-coding RNA) has been suggested to alleviate the progression of osteoporosis. Chen et al. found that lncRNA BMNCR expression was decreased in the bone marrow and spleen of osteoporotic mice [57]. Specifically, its expression was decreased during RANKL-induced osteoclast differentiation [57]. This would suggest that lncRNA BMNCR helps inhibit osteoporotic change.

#### 2.2.3. lncRNA CASC11

lncRNA CASC11 (cancer susceptibility 11) has been shown to be upregulated in PMOP [58]. A small study consisting of blood samples from 67 patients with PMOP showed that CASC11 and TNF-α were both increased in their plasma [58]. The researchers deduced that overexpression of CASC11 led to TNF-α upregulation in osteoclasts [58]. Furthermore, plasma levels of CASC11 and TNF-α were found to be decreased after treatment with elcatonin [58]. Higher CASC11 levels were also associated with a prolonged treatment period [58]. However, the molecular mechanism by which CASC11 regulates TNF-α remains unknown. For now, the measurement of plasma levels of CASC11 may indicate which patients may need a longer treatment course for PMOP.

#### 2.2.4. lncRNA CRNDE

The lncRNA colorectal neoplasia differentially expressed (CRNDE), first identified in colorectal tumors, is found to be increased in the osteoclasts of postmenopausal women compared to healthy women in a study by Li et al. [10]. Overexpression of CRNDE in osteoclasts of healthy women improved cell proliferation rate, while CRNDE knockdown in osteoclasts of osteoporotic women inhibited cell proliferation. In addition, cell percentage declined in the S-phase during CRDNE knockdown compared to overexpression, causing apoptosis [10]. The study inferred that CRNDE played a role in regulating cell apoptosis, and knockdown could halt the proliferation of osteoclasts.

#### 2.2.5. lncRNA GAS5

Low levels of lncRNA GAS5 (growth arrest-specific 5) have been found in bone tissue of patients with PMOP [59]. Feng et al. showed that GAS5 could regulate the expression of RUNX2 through mRNA-498, which negatively regulates osteogenic differentiation [59]. Thus, overexpression of GAS5 could halt the progression of osteoporosis.

#### 2.2.6. lncRNA MALAT1

The lncRNA metastasis-associated lung adenocarcinoma transcript 1 (MALAT1), also known as nuclear-enriched transcript 2 (NEAT2), is involved in osteoporosis, as well as serving as a prognostic biomarker for lung cancer metastases [60]. However, studies have shown conflicting results in the mouse model. Yang et al. concluded that exosomal MALAT1 derived from BMSCs could afford to enhance osteoblastic activity and improve symptoms of osteoporosis [60]. Zheng et al., on the other hand, found that MALAT1 inhibited osteogenic differentiation of BMSCs through the enhancement of the MAPK signaling pathway, and promoted the progression of osteoporosis [61]. Further studies are required to clarify these findings.

#### 2.2.7. lncRNA MEG3

The lncRNA MEG3 (maternally expressed 3) was found to promote osteoporosis in non-cancerous subjects [62]. In both ovariectomized mice and women with PMOP, expression of miR-133a-3p and MEG3 were found to be significantly higher in bone tissue compared to controls [62]. There was a positive correlation between miR-133a-3p and MEG3 expression in BMSCs; MEG3 overexpression significantly increases miR-133a-3p expression by direct binding, downregulating osteogenic differentiation [62,63]. On the other hand, MEG3 has been found to play a critical role in osteoblastic differentiation in the treatment of multiple myeloma [9], suggesting disease-dependent effects and calling for further studies to clarify these findings.

#### 2.2.8. lncRNA MSC-AS1

lncRNA MSC-AS1 (MSC antisense RNA 1) may alleviate osteoporosis. Expression of MSC-AS1 was found to increase with osteogenic differentiation of mice BMSCs, as well as osteogenesis-related genes, such as RUNX2, osteopontin, and osteocalcin [64]. Knockdown of MSC-AS1, on the other hand, downregulated BMP2, p-smad1/5/8, and RUNX2 [64]. The above findings suggest that MSC-AS1 plays a role in inducing osteogenic differentiation, thus alleviating osteoporosis.

#### 2.2.9. lncRNA NEF

Plasma levels of lncRNA NEF (neighboring enhancer of FOXA2) are downregulated in PMOP women [65]. In addition, this study found that low plasma levels of lncRNA NEF were significantly correlated to a longer treatment course of elcatonin until BMD returned to normal range (which was within three months for all patients). lncRNA NEF may interact with IL-6 to produce these effects [65] and can be used as a biomarker of the disease.

#### 2.2.10. lncRNA SNHG1

lncRNA SNHG1 (small nucleolar RNA host gene 1) has been found to be downregulated in osteoporosis [66]. Compared with healthy postmenopausal women, postmenopausal women with osteoporosis had lower plasma levels of SNHG1 in a 6-year follow-up study [66]. The study also found that anti-osteoportic treatment, such as bisphosphonates and hormone replacement therapy, could upregulate plasma SNHG1 [66]. Although SNHG1 is well-established in cancer biology as a regulator of cancer cell behavior [66], the molecular mechanism of SNHG1 in osteoporosis is still unknown. It is known that SNHG1 is involved with multiple miRNAs, such as miR-145, miR-195, miR-338, and miR-497, which are involved in the differentiation of osteoblasts and osteoclasts [66]. Therefore, SNHG1 may potentially be used as a biomarker for both the diagnosis and treatment for PMOP.

#### 2.2.11. lncRNA TUG1

lncRNA TUG1 (taurine-upregulated gene 1) is thought to be upregulated in osteoporosis. This theory is based on the fact that it is inhibited in ankylosing spondylitis, which is commonly thought of as an inverse pathological process to osteoporosis [67]. In the plasma of patients of both genders at various stages of osteoporosis, there was upregulation of lncRNA TUG1 [67]. Although the mechanism of action of lncRNA TUG1 has not yet been elucidated, it is thought to sponge miR-204-5p to promote osteoblast differentiation [67]. It is, therefore, a potential diagnostic marker for osteoporosis.

#### 2.2.12. lncRNA XIXT

lncRNA XIXT (X-inactive specific transcript), which could potentially alleviate osteoporosis, was found to be downregulated in the serum of osteoporotic patients [68]. It was found to promote osteogenic differentiation of BMSCs and to halt osteoporosis progression through targeting miRNA-30a-5p [68]. In addition, knockdown of miRNA-30a-5p enhances the expression of RUNX2, and vice versa, suggesting RUNX2 is the downstream target of miRNA-30a-5p [68]. Therefore, XIXT could be a potential novel therapeutic target for PMOP.

## 3. Discussion

The field of miRNAs and lncRNAs with regards to osteoporosis is still relatively new, and this is reflected in the quantity of research currently available. Often, there are conflicting results in the literature, such as miR-223 affording to both promote and inhibit osteoclastogenesis [3]. Wijnen et al. suggest that miRNAs provide both positive and negative cross-talk between different regulatory pathways [3], thereby leading to this phenomenon. Another possible explanation is that miRNA is present in different clinical specimens. For example, Mandourah et al. found that, while both miR-122-5p and miR-4516 were suitable biomarkers for osteoporosis, miR-122-5p was detectable in the serum, while miR-4516 was found in the plasma [33]. A large cohort study of 682 women found that there was a lack of association between bone parameters and circulating levels of miRNAs, stating results were canceled out after age adjustment [69]. The authors suggest this could be due to the fact that age was also strongly correlated with the serum levels of the 32 miRNAs they selected [69]. On the other hand, another pilot study suggests that the combination of many miRNAs can help predict fragility fracture risk [70]. Perhaps it makes sense that one cannot look at each miRNA in isolation, as the cellular processes of OP work in a synergistic fashion. Finally, many studies on miRNA deregulation lack control groups [7]. These factors are worth bearing in mind when conducting future clinical trials.

The current review of literature was retrieved from studies of different sources (human and animals) and tissues (bone cells, serum, and tissue fluid), which may explain the many conflicting results. Thus, conflicting conclusions have been reported for most miRNAs and lncRNAs depending on cellular models used, animal studies or cohorts of humans, and even analytical methods. We have specified these sources and differences where possible. One of the advantages of animal studies is the convenience in conducting these studies. Some animals (such as mice) have a shorter growth cycle than that of a human being and are subject to investigations in a limited research period. Furthermore, gene knockout can be implemented in an animal model to investigate the depletion effect of genes, DNAs, as well as RNAs on the target organs, which cannot be performed in a human being. However, the major disadvantage of animal studies lies in the differences of genes and subsequent RNAs between animals and human beings, and thus generalization of the conclusions of animal studies to human beings is limited. In contrast, the results of human studies are observational but direct evidence. The major disadvantage of human studies is that intervention (such as gene knockout) cannot be implemented due to the consideration of moral hazards. In addition, the expression of genes, miRNAs, or lncRNAs in variant tissues (bone cells, serum, and tissue fluid) may be different, thus resulting in conflicting conclusions.

Due to the lack of clear-cut associations between PMOP and the expression of miRNAs and lncRNAs at present, there is still a long way to go before they can be used as potential noninvasive biomarkers. In addition, there are also many barriers still to overcome to transfer miRNA and lncRNA knowledge to the synthesis of clinical therapeutic drugs. Synthetic oligonucleotides mimicking miRNAs have a limited half-life due to degradation by nucleases in the bloodstream, and they also have a poor capacity to penetrate host cell membranes to reach their target cells [71]. lncRNA degrades even more easily than miRNA due to their low structural stability [9]. At present, antagomiRs, viruses, scaffold-based miRNA delivery, and extracellular vesicles have all been used as vectors in miRNA studies [71]. AntagomiRs are direct mRNA inhibitors, but, unfortunately, are required at a high dose to work [71]. Adeno-associated viruses are small vectors non-pathogenic to humans but are expensive [71]. Scaffold-based miRNA delivery provides not only structural support but also a convenient environment for bone tissue growth [71]. Finally, extracellular vesicles are natural bioabsorbable gene carriers that can recognize target cells, with the great advantages of oral administration and ease of long-term storage [71]. There are also currently no reports of lncRNAs in current osteoporosis treatment up to date.

## 4. Conclusions

In conclusion, miRNAs and lncRNAs are two potential targets that are the logical next step in osteoporosis research. Further research into the epigenetic modification and the regulatory roles of these molecules will bring us closer to potential disease-modifying treatment for PMOP. This review provides current opinions on the roles of miRNAs and lncRNAs in osteoporosis. However, more issues regarding the detailed actions of miRNAs and lncRNAs in osteoporosis remain unknown and controversial and warrant future investigation.

## Figures and Tables

**Table 1:** List of micro RNAs (miRNAs), their actions, and expression in postmenopausal osteoporosis (PMOP). Studies were performed in humans unless otherwise stated in parentheses.

miRNA	Action	Expression in PMOP	Sources	References
miR-9-5p	inhibit osteogenesis, promote adipogenesis promote osteoclastogenesis	high	serum	Zhang et al. [14] Wang et al. [15]
miR-21	promote osteoclastogenesis promote osteogenesis and inhibit osteoclastogenesis	unclear		Jiang et al. [16] Cheng at al. [17] Seeliger et al. [18] Yavropoulou et al. [19] Yang et al. [23] Hu et al. [24]
miR-29	unclear	low	serum	Tang et al. [1] Lian et al. [25] Kocijan et al. [26] Li et al. [27] Kapinas et al. [28] Rossi et al. [29] Bottani et al. [31]
miR-30b-5p	negatively regulate osteoblast differentiation	low	serum	Bottani et al. [31]
miR-31	unclear	low	bone	Tang et a. [1] Foessl et al. [5] Mäkitie et al. [32]
miR-100	inhibit osteogenic differentiation	high	bone and serum	Cheng at al. [17] Seeliger et al. [18]
miR-103-3p	inhibit osteoblast differentiation and proliferation	low	serum	Bottani et al. [31]
miR-122-5p	inhibit osteoblast differentiation	low	serum	Mandourah et al. [33]
miR-124	inhibit osteoclast formation inhibit osteogenesis	high	serum	Tang et al. [1] Yavropoulou et al. [19] Qadiret et al. [34]
miR-133	inhibit osteoblast differentiation increase osteoclastogenesis	high	bone and serum (mouse)	Tang et al. [1] Cheng at al. [17] Wang et al. [35] Kocijan et al. [36]
miR-135a-5p	inhibit osteogenic differentiation	high	bone	Shi et al. [37]
miR-146a	inhibit osteoclastogenesis	high	bone (mouse)	Tang et al. [1]
miR-148a	induce osteoclast formation	high	Serum (mouse)	Tang et al. [1] Xiao et al. [38]
miR-155	regulate osteoclastogenesis	high	unclear (mouse)	Tang et al. [1]
miR-182-5p	inhibited ADCY6 expression and Rap1/MAPK signaling pathway activation	high	bone and serum (mouse)	Pan et al. [39]
miR-194-5p	unclear	high	whole blood lysate	Foessl et al. [5]
miR-200a-3p	inhibit osteogenic differentiation	high	serum	Lv et al. [40]
miR-203a	slow osteoblast differentiation	high	bone	Kocijan et al. [36]
miR-214-5p	promote adipogenic differentiation	high	(in vitro)	Qiu et al. [41]
miR-221	inhibit osteogenic inhibition	low	bone	Zhang et al. [42]
miR-223	inhibit osteoclast differentiation	unclear	serum	Tang et al. [1] Seeliger et al. [18] Pickering et al. [43]
miR-338	regulate osteoblast differentiation	high	serum	Guo et al. [44] Lin et al. [35]
miR-365	suppresses MMP-9	low	bone (mouse)	Li et al. [46]
miR-410	regulate BMP-2 expression	high	serum	van Wijnen et al. [3] Zhang et al. [47]
miR-422a	may stimulate osteoclastogenesis	high	human circulating monocytes	Bottani et al. [31] Cao et al. [48]
miR-449b-5p	inhibit osteogenic differentiation	unclear	(in vivo)	Li et al. [49]
miR-503	inhibit osteoclast differentiation	low	human circulating monocytes	Tang et al. [1]
miR-543	promote osteoblast apoptosis	high	bone (mouse)	Li et al. [50]
miR-579-3p	inhibit osteogenic differentiation	high	serum	Luo et al. [51]
miR-874	promote osteoblast proliferation	low	bone (mouse)	Lin et al. [52]
miR-1297	inhibit osteogenic differentiation	high	bone	Wang et al. [53]
miR-2861	promote osteoblast differentiation	high	serum	Yavropoulou et al. [19] Li et al. [54]

**Table 2:** List of long-non-coding RNAs (lncRNAs), their actions, and expression in PMOP. Studies were performed in humans unless otherwise stated in parentheses.

lncRNA	Action	Expression in PMOP	Sources	References
ANCR	inhibit osteoblasts, increase osteoclastogenesis	high	blood mononuclear cells	Wu et al. [9] Cai et al. [55] Tong et al. [56]
BMNCR	inhibit osteoporosis	low	bone (mouse)	Chen et al. [57]
CASC11	lead to TNF-α upregulation in osteoclasts	high	plasma	Yu et al. [58]
CRNDE	regulate cell apoptosis	high	bone	Li et al. [10]
GAS5	regulate osteogenic differentiation	low	bone	Feng et al. [59]
MALAT1	unclear	low	bone (mouse)	Yang et al. [60] Zheng et al. [61]
MEG3	unclear	high	bone	Wu et al. [9] Wang et al. [62] Sun et al. [63]
MSC-AS1	induce osteogenic differentiation	unclear	bone (mouse)	Zhang et al. [64]
NEF	interact with IL-6	low	plasma	Ma et al. [65]
SNHG1	unclear	low	plasma	Huang et al. [66]
TUG1	may promote osteoclast differentiation	high	plasma	Han et al. [67]
XIXT	promote osteogenic differentiation of BMSCs	low	Serum	Zhang et al. [68]

## Abbreviations

3-UTR	3-untranslated regions
ADCY6	adenylate cyclase 6
AKT	phosphorylated-protein kinase B
ALP	alkaline phosphatase
BMD	bone mineral density
BMSC	bone mesenchymal stem cell
BMP-2	bone morphogenetic protein 2
COL1A1	collagen α-1 (I) chain
COL4A1	collagen type IV α1 chain
CXCL12	C-X-C motif chemokine ligand 12
DElncRNAs	differentially expressed lncRNAs
DEmRNAs	differentially expressed mRNAs
ERα	estrogen receptor alpha
GIOP	glucocorticoid-induced osteoporosis
HDAC5	histone deacetylase 5
lncRNA	long non-coding RNA
MAPK	mitogen-activated protein kinase
mRNA	messenger RNA
miRNA	micro RNA
MITF	microphthalmia-associated transcription factor
MMP-9	matrix metalloproteinase-9
PI3K	phosphoinositide-3-kinase regulatory subunit 1
PMOP	postmenopausal osteoporosis
RANKL	receptor activator of nuclear factor-κB ligand
RNA	ribonucleic acid
RT-qPCR	real-time quantitative polymerase chain reaction
RUNX2	runt-related transcription factor 2
SATB2	special AT-rich sequence-binding protein 2
SOX4	SRY-box transcription factor 4
SUFU	suppressor of fused gene
TGF	transforming growth factor
YAF-2	YY1-associated factor 2
